# A Physician's Anthology

**Published:** 1921-04-02

**Authors:** 


### A Physician's Anthology.

The Oxford University Press has a long series of admirable anthologies to its honour, but the latest, selected by Dr. Casey A. Wood and Dr. Fielding H. Garrison, is something new. Intended originally for "a birthday present to the late Sir William Osler by one of his old Montreal students and by another American 'pupil,'" the collection was read and approved by him before his death fifteen months ago. The introduction records the impressions that Osler made upon his colleagues who are most entitled to speak, and then tells us that, though he never wrote verse, he encouraged, through his conversation and writings, the study of English poetry. To this extent, then, he is in the tradition of Thomas Lodge, Sir Thomas Browne, Mark Akenside, and the present Poet Laureate. But, Dr. Garrison remarks, "the effect of medical training upon the individual is peculiar, in that it frequently gives a materialistic bias to the mind. But the effect of medical experience is different." It is the reflection of the latter in lyric poetry that the reader finds in this collection, from which devotional and erotic verse is omitted. The principal charm to us is that most of those poems which occur in ordinary anthologies have been left out; and the reader turns with pleasure to a delightful lyric by Pope, to several engaging (if not inspired) poems by W. E. H. Lecky, to Emily Brontë's impassioned lyrics, and even, such is Oxford scholarship, to the famous verse by Thomas Osbert Mordaunt, which, till only the other day, was attributed to Walter Scott. Matthew Arnold and Clough are also well represented; and, in the main, by reading the book we have extended our knowledge of English verse rather than re-read yet again its familiar masterpieces. "Of those who walk alone," by Richard Burton, is one of the poems which deserves to be better known than it is; and the subjects into which the poems are divided give just that hint for the satisfaction of a mood which best beguiles the attention of the reader. We hope that the volume will find a welcome, and are glad that Osler provided the excuse for it. At the modest price of 8s. 6d. net, Mr. Milford has provided an interesting pocket volume which deserves the care that has been spent upon it.

